# iPads in early education: separating assumptions and evidence

**DOI:** 10.3389/fpsyg.2014.00715

**Published:** 2014-07-08

**Authors:** Natalia Kucirkova

**Affiliations:** Faculty of Education and Language Studies, The Open UniversityMilton Keynes, UK

**Keywords:** iPads, early literacy, apps, early education, design-based research

Since their first appearance in 2010, iPads and other comparable tablets have been heralded for their potential to revolutionize education, including that of young children. Like previous multimedia technologies (e.g., whiteboards, Kozma, 1991), iPads are multimodal, allowing users to use texts, pictures, and sounds. In comparison with other, so far available multimedia technologies, iPads have three novel features which have the potential to make a positive difference to early education: iPads are portable and light-weight (unlike netbooks and laptops), they eliminate the need for separate input devices requiring certain levels of dexterity (such as mouse and keyboard) and thirdly, they are specifically designed to accommodate a number of apps, many of which have a child-friendly intuitive design. With several of these apps, iPads provide unprecedented opportunities for children to create their own contents and participate in rich and dynamic learning contexts.

Yet, despite the possible benefits, there is an absence of research supporting the enthusiastic claims that iPads will “revolutionize education” (e.g., Ferenstein, 2011). This is due to several reasons but in early education, two prevalent myths concerning new technologies hinder research progress and innovation in practice: technological determinism and the digital/non-digital binary. This article outlines how these technology myths relate to the emerging iPad research in early education of children aged between 2 and 8 years old. After a critical assessment of the assumptions underlying some of the studies, attention is turned to a “second wave” of iPads' research which avoids these conceptual obstacles. Recommendations for future research are provided throughout the article, with the aim of provoking a wider debate regarding some of the identified issues.

## The first wave of iPad research

Over the past decade, two myths have arisen with regard to technologies in early years. One dominant misunderstanding concerns the troubled relationship between digital and non-digital resources (e.g., digital vs. paper books) used with young children (Kucirkova, 2014). The consequence of this myth leads to research and practice which position technology and traditional resources in opposition, rather than in complementary relation to each other (Edwards, 2013). The second popular misconception is related to technological determinism (Livingstone et al., 2013) which positions technology as the driving force for educational change, without recognizing the powerful role of context and individual variables which shape technology deployment in the early years.

Both myths could be seen as being propagated with the first wave of iPad research. First, there is an emerging body of research which aims to compare the learning benefits of iPads with printed text resources. Notably in relation to book reading with young children, researchers have studied the effects of interactive books presented in iPad format against printed texts. In one such comparative study, Chiong et al. (2012) observed thirty-two parents and their 3–6-year old children to read books in three formats: printed books, simple and enhanced e-books (the last one to represent iPad books). The findings indicated that iPad books prompted more non-content related interactions in both parents and children. Different results were obtained in a study with twenty primary school children aged 11–12. Dundar and Akcayir (2012) compared children's reading speed and comprehension when reading a printed and iPad book. The research found no significant difference between children who read the texts on tablets or in paper. In another study, Masataka (2014) compared the effects of intensive exposure to iPads and printed picture book in relation to the reading ability of 4-year old Japanese boys. The author found an increase after children's exposure to the iPad book but not the printed book. The interpretation of these findings is problematic because the studies do not separate out effects of methods with the effects of different formats or media (cf. Sung and Mayer, 2013). Children use different technologies for different purposes, as recently confirmed in a nationwide survey by Booktrust and Pearson (O'Donnell and Hallam, 2014). In the Read for My School Competition, 7–13-years old children were given the opportunity to read books online or offline. A survey of more than 1000 children found that 77% of children prefer to access the books in digital format (on e-readers or tablets). However, 68% also said that they would prefer to read “a good story” in a printed format. These seemingly contradictory responses show that like-with-like comparisons between iPads and printed texts are difficult to interpret because each format comes with different affordances, corresponding to different intents. This is also supported by a recent study by Krcmar and Cingel (2014) who compared iPad and traditional books in relation to parent-child interaction and child's reading comprehension. The authors found children's increased comprehension in the traditional book format but this was closely related to parents' increased distraction talk when sharing the iPad book. The interrelationship between books' content and format and their joint impact on the context of book reading is necessary to take into account in evaluative studies. Therefore, to better understand children's preferences and reading behavior with iPad books, future research should adopt a dynamic evaluative framework instead of a comparative design.

This recommendation applies also to another problematic issue in iPad research, namely the panacea role assigned to technology in public schools. In the past few years, large-scale iPad projects have resurfaced in several countries worldwide, including Turkey (The FATIH Project), USA (e.g., The LAUSD project in Los Angeles), UK (e.g., iPad Scotland), or Australia (Department's iPads for Learning Trial). From the technological determinism-critical perspective, such projects may be criticized for a “top-down” approach where an intervention has been implemented before it has been formally evaluated. There is a tendency to perceive technology, including iPads, as a quick fix solution for outstanding educational problems, without giving due consideration to the idiosyncrasies of individual educational contexts. Furthermore, a common criticism of the programs is that they provide hardware without software which would support curriculum material, and that professional development is minimal or non-existent. Such a modus operandi is unlikely to generate an educational revolution or change in practice. Rather, it is likely to reproduce dominant models of instruction. Not surprisingly, pioneering studies (see e.g., http://www.ipadsforeducation.vic.edu.au/ipad-student-trial/ipads-in-schools) show that it is the pedagogy contextualizing the use of iPads rather than the device *per se* that makes a difference to children's learning.

This linkage between pedagogy, or ethos of the school, and iPads' deployment, has been documented in a number of early observational studies. Hutchison et al. (2012) found that the learning potential of iPads is directly related to the teachers' ability to effectively leverage iPads' affordances and creatively link them to the curriculum. Similarly, Flewitt et al. (2014) showed that there was considerable variability in the ways iPads were used across three settings (Children's Centre nursery, a primary school Reception class and a Special School) but that well-planned, iPad-based literacy activities stimulated a number of positive attitudes and behaviors in children in all three contexts. Such findings remind us that technology can at times be “a replaceable element in the picture” (Crook and Lewthwaite, 2013, p. 439) and that we “need to appreciate the systemic and institutional nature of educational practice” (Crook and Lewthwaite, 2013, p. 457). How would these findings be different if iPads were replaced with for example portable laptops or say digital cameras? Teachers' and children's own competence with the devices, and the social, political, religious, cultural influences, and other factors of the instructional context need to be factored in when interpreting the effects of iPad use in observational studies. This “broader context of classroom discourse” (Crook, 1991, p. 81), is intimately linked to the ways in which iPads support learning.

## Second wave of iPads research and future avenues

Future research needs to critically examine the potential of iPads to act as an innovative pedagogical support to current classroom practices and instructional strategies. This can be achieved with well-established and robust methodologies such as randomized controlled trials (RCTs) or design-based research (DBR). RCTs, though having some challenges, are the “gold standard” (Cook, 2009, p. 1), for identifying the cause-effect relationship between an intervention (such as for example use of iPads in a classroom) and outcome (such as for example children's increased learning scores). DBR aims to generate “new theories, artifacts, and practices that account for and potentially impact learning and teaching in naturalistic settings” (p. 2, Barab and Squire, 2004) and as such, can provide deeper theoretical insights and realistic understanding of the learning potential.

In a recent RCT on the Masamu tablet intervention in Malawi, Pitchford (2014) found that iPads can raise mathematical standards. After an 8-week intervention programme, 78% of low achieving children showed improved mathematics skills, compared to a 17% increase in children who received Normal Practice. This shows that in contexts where there is a shortage of qualified and effective teachers, iPads can be a cost-effective means of delivering individualized, up-to-date learning content which is aligned with innovative teaching practices.

Another promising avenue for future research is the possibility of using innovative methodologies which invite co-participation of teachers and iPad software designers. Falloon's (2013a) employed a new digital analysis method to examine in detail the design and content features of a number of literacy, numeracy and problem-solving apps used by 5-year olds. In further publications drawing on findings derived from the same study group, Falloon (2013b) and Falloon and Khoo (2014) detailed how young children engaged in problem-solving using specific apps and how their own dispositions and app design together affected the kinds of interactions he observed.

Furthermore, with DBR, researchers can examine children's interactions with apps they have (co-) produced and where researchers employ mixed methods. This provides unique insights and greater awareness of the studied phenomena. In our study (Kucirkova et al., 2014), we studied children's interactions with a story-making app we designed (OS), and a set of commercially produced drawing and puzzle-making apps. The OS app has been developed through an iterative design process, with a close study of the instructional strategies and tools used in the classroom. Children's interactions were studied qualitatively and quantitatively and in relation to their use of exploratory talk which is indicative of effective classroom discourse. We found that children's use of exploratory talk was similar with apps which support joint problem-solving, open-ended content production and which have incrementally difficult tasks to solve. It was the unique insight provided through DBR and a careful documentation of the software-hardware relationships that allowed us to inform educators and policy-makers on the innovative features introduced by iPads.

## Conclusion

Research to date has provided exploratory insights into the affordances and nature of children's interactions with iPads in early years classrooms. The first wave of iPad research describes many educational benefits which are yet to be realized and formally evaluated. Carefully designed studies which draw on DBR or RCTs can challenge some common assumptions on the role of technology in early years and derive meaningful results with direct pedagogical applications and intervention justifications.

### Conflict of interest statement

Natalia Kucirkova currently works as a Knowledge Transfer Partnership Associate for Booktrust and The Open University.
